# Survey of Intraocular Antibiotics Prophylaxis Practice after Open Globe Injury in China

**DOI:** 10.1371/journal.pone.0156856

**Published:** 2016-06-08

**Authors:** Bingsheng Lou, Lixia Lin, Junlian Tan, Yao Yang, Zhaohui Yuan, Xiaofeng Lin

**Affiliations:** State Key Laboratory of Ophthalmology, Zhongshan Ophthalmic Center, Sun Yat-sen University, Guangzhou, China; Massachusetts Eye & Ear Infirmary, Harvard Medical School, UNITED STATES

## Abstract

**Purpose:**

To elucidate the Chinese practice of intraocular antibiotics administration for prophylaxis after open globe injury.

**Methods:**

A cross-sectional questionnaire survey was performed online by scanning a Quickmark (QR) code with smartphones at the 20^th^ Chinese National Conference of Ocular Trauma in November 2014.

**Results:**

A total of 153 (30.6%) of all participators at the conference responded. Of the respondents, 20.9% were routinely administered with prophylactic intraocular injection of antibiotics at the conclusion of the primary eye repair, and 56.9% were used only in cases with high risk of endophthalmitis development. The intraocular route of delivery was mainly included with intracameral injection (47.9%) and intravitreal injection (42.0%). Cephalosporins (53.8%) and vancomycin (42.0%) were the main choices of antibiotic agents, followed by fluoroquinolones (24.3%), and aminoglycosides (13.4%). Only 21.9% preferred a combination of two or more two drugs routinely. In addition, significantly more respondents from the referral eye hospital (92.7%) replied using intraocular antibiotics injection for prophylaxis compared to those respondents from the primary hospital (69.4%) (*p* = 0.001, Fisher’s exact test).

**Conclusions:**

Intraocular antibiotics injection for post-traumatic endophthalmitis prophylaxis is widely used in China. However, the choice of antibiotic agents and the intraocular route of delivery vary. A well-designed clinical trial is needed to establish a standardized protocol of intraocular antibiotics administration for post-traumatic endophthalmitis prophylaxis.

## Introduction

Endophthalmitis is the most devastating complication after open globe injury.[1] As previously reported, the incidence of endophthalmitis following open-globe injury ranges from 0.9% to 30% in different studies,[2–7] which is higher than the incidence of post-operative endophthalmitis (0.03%-0.68%) [8–12]. Despite aggressive application of antibiotics and prompt surgical intervention, irreversible retinal damage and severe visual loss still occur, with only 22% to 42% of patients obtaining a final visual acuity better than 20/400 [8, 13, 14]. These findings indicate that the appropriate prophylactic strategies initiated before endophthalmitis development are crucial. However, there is no general consensus among the eye clinicians regarding the best prophylactic guidelines for post-traumatic endophthalmitis. Traditionally, prophylactic intravenous and topical antibiotics have been commonly administered in most cases of open globe injuries, although they are not well supported by strong experimental evidence and randomized prospective clinical trials.

Due to the poor ocular penetration of topical and systemic antibiotics, some eye clinicians advocate the use of intraocular antibiotics in prophylaxis at the end of primary eye repair, which has been increasingly used for prophylaxis within the last decade [15, 16]. Although the administration of intraocular antibiotics has been preliminarily proven to decrease the risk of endophthalmitis in cases of open globe injuries with intraocular foreign bodies (IOFBs) in previous studies,[15, 17, 18] with lacking of general guidelines to suggest any appropriate type of antibiotics and any route of intraocular administration, not all clinicians agree with the routine administration of intravitreal antibiotics in prophylaxis [1, 8, 19, 20]. Theoretically, prophylactic intraocular antibiotics may increase the risk of antimicrobial resistance development, the retinal toxicity of antibiotics and complications of the operation itself. In addition, if suprachoroidal hemorrhage or retinal detachment is present, then the administration of antibiotics into the eye could result in the injection of drug inadvertently into the suprachoroidal space or subretinal space. Thus, the role of intraocular antibiotics as a routine prophylaxis against endophthalmitis has not been well verified by a larger randomized prospective study with long follow-up.

It has been demonstrated that eye trauma is the main cause (72.1%) of infectious endophthalmitis in China [21]. However, in the developed countries, post-operative endophthalmitis takes a dominant role, and post-traumatic endophthalmitis comprises only 25–31% of all cases [1, 8, 22]. China is a developing country dominated with labor-concentrated industries. The predominant component of post-traumatic endophthalmitis in infectious endophthalmitis might mainly result from the industrial structure and weak occupational protection in China. In addition, the poor medical and health service system may also play a role.

How to minimize the development of endophthalmitis after open globe injures, it is necessary to establish appropriate guidelines in prophylaxis against endophthalmitis. Unfortunately, there is an absence of information on the practice patterns of prophylaxis endophthalmitis after open globe injuries in China. In the current survey, we intended to explore the practice patterns of intraocular antibiotics in prophylaxis against endophthalmitis after open globe injuries and clarify the national trend in China.

## Materials and Methods

This study was a cross-sectional questionnaire survey performed at the 20^th^ Chinese National Conference of Ocular Trauma, which was held on the 30^th^ of October to the 2^nd^ of November in 2014. The conference aimed at academic communication for nation-wide ocular trauma specialists and ophthalmologists interested in ocular trauma in China. The introduction of this survey was posted at the conference registration hall and the lecture hall. Participants voluntarily accessed an online survey by scanning the Quickmark (QR) code with their smartphone, where each smartphone could only answer the questionnaire once. An assistant was arranged to further introduce how to access the online questionnaire for the participants. The survey consisted of 8 questions, which required approximately 2 minutes to accomplish. The questionnaire was shown in the S1 File. The study adhered to the tenets of the Declaration of Helsinki. The waiver of written informed consent and study protocol was approved by the Institutional Review Board of the Zhongshan Ophthalmic Center at Sun Yat-Sen University in China. The survey was explained in detail that it was voluntary and would not influence care. The data were collected and analyzed anonymously. The chi-square test or Fisher’s exact test was performed to compare the differences about the intraocular antibiotics prophylaxis practice between in the referral eye hospital and in the primary hospital or between less than 50 cases group and more than 50 cases group of the annual volume of primary eye repair surgery. A 2-tailed *p*-value less than 0.05 was considered statistically significant. Statistical analyses were performed using the software SPSS (Version 20.0, SPSS Inc, Chicago, IL, USA).

## Results

A total of 500 Chinese ophthalmologists were registered at the 20^th^ National Conference of Ocular Trauma, of whom 153 (30.6%) responded. All respondents indicated that they had managed the patients of open globe injuries, of whom 70 (45.8%) performed more than 50 cases of the expected average annual volume of primary eye repair (Table 1).

**Table 1:** Distribution of respondents by annual eye repair volume.

Volume (cases)	Respondents (n)
0	0
<50	83 (54.2%)
50–100	44 (28.8%)
>100	26 (17.0%)
Total	153

Only 32 respondents (20.9%) routinely administered prophylactic intraocular injection of antibiotics at the conclusion of the primary eye repair, and 34 respondents (22.2%) had never used the medication. However, 87 (56.9%) of the respondents used the intraocular injection of antibiotics only in cases with high risk of endophthalmitis development, such as rupture of the lens capsule, intraocular foreign body remains, delayed primary closure of the wound >24 hours, injury in a dirty environment, large wound and obvious inflammation reaction before wound repair (Table 2).

**Table 2:** Prophylactic intraocular injection of antibiotics at the end of primary eye repair.

Use of prophylactic intraocular antibiotics	Respondents (n)
Routinely use	32 (20.9%)
Use, depending on different conditions	87 (56.9%)
Rupture of lens capsule	19
Intraocular foreign body	54
Wound repair delayed > 24 hrs	42
Injured in dirty environment	54
Serious inflammation reaction	52
Large wound	29
Not use	34 (22.2%)
Total	153

**Fig 1:**
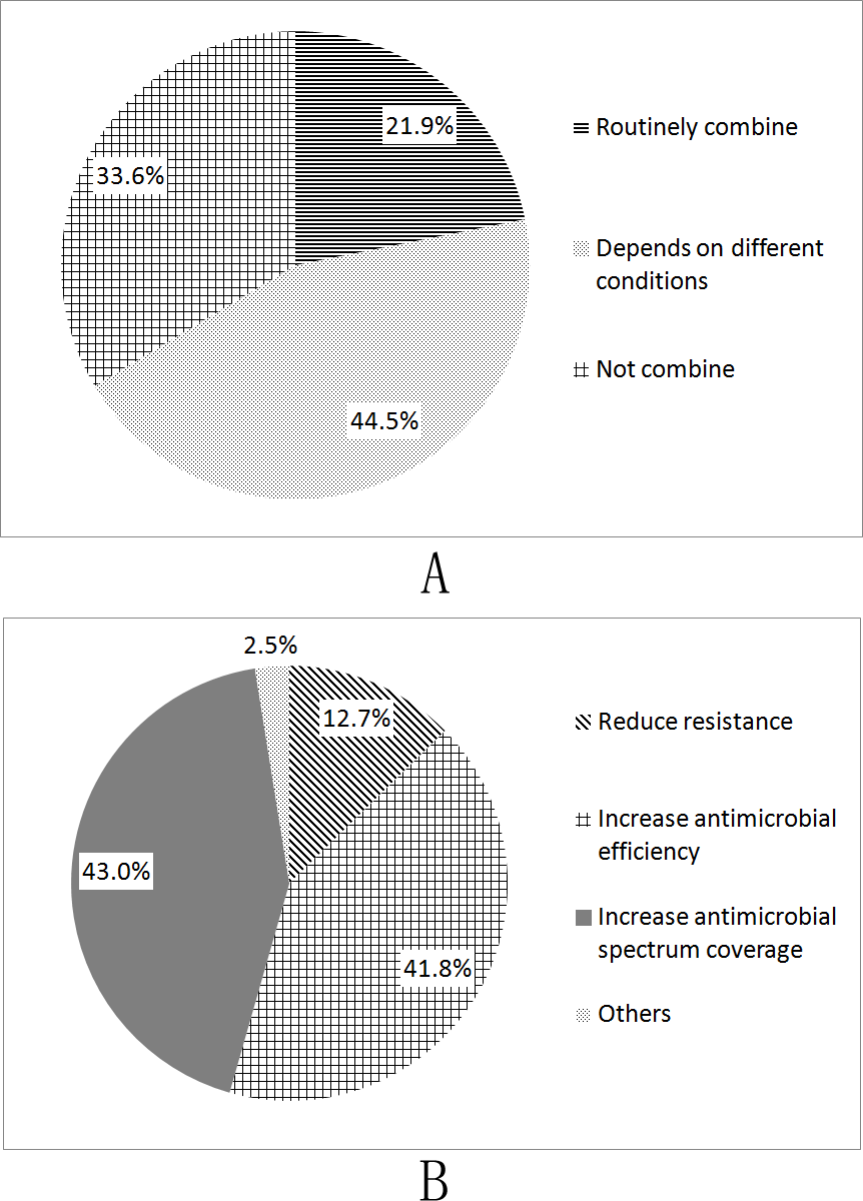
Response to combination of intraocular antibiotic agents. (A). Respondents using a combination of two or more antibiotic agents for intraocular administration. (B). Reasons for combination of intraocular antibiotics.

With the decision to use intraocular injection of antibiotics, 47.9% reported the use of intracameral injection, followed by intravitreal injection (42.0%). In addition, 10.1% responded to the administration of antibiotics via the wound (Table 3). The choice of antibiotic agents for intraocular injection is shown in Table 4. Furthermore, 64 respondents (53.8%) preferred to use cephalosporins, followed by vancomycin (42.0%), fluoroquinolones (24.3%), and aminoglycosides (13.4%). In addition, most of the respondents (21.9% routinely and 44.5% depended on the conditions of patients) responded using a combination of two or more antibiotic agents for intraocular injection. However, 33.6% of the respondents used only a single antibiotic agent routinely (Fig 1A). The main reasons cited for the combined application of antibiotics were to increase the antimicrobial spectrum coverage (43.0%) and antimicrobial efficiency (41.8%) (Fig 1B).

**Table 3:** Intraocular route of antibiotic agents delivery in open globe injuries.

Route of delivery	Respondents (n)
Intracameral injection	57 (47.9%)
Intravitreal injection	50 (42.0%)
Injection via wound	12 (10.1%)
Total	119

**Table 4:** Choice of antibiotics for intraocular administration.

Antibiotics	Respondents (n)
Fluoroquinolones	29 (24.3%)
Cephalosporins	64 (53.8%)
Aminoglycosides	16 (13.4%)
Vancomycin	50 (42.0%)
Others	3 (2.5%)
Total	119

The selection of the appropriate type of antibiotics for endophthalmitis prophylaxis is based on knowledge of the bacterium spectrum in post-traumatic endophthalmitis. In our survey, only 85 respondents (55.6%) responded with Gram-positive *cocci* as the most common causative pathogen in post-traumatic endophthalmitis. Moreover, 21 respondents (13.7%) reported having no idea about the bacterium spectrum in post-traumatic endophthalmitis (Table 5).

**Table 5:** The most common causative pathogen in post-traumatic endophthalmitis.

Pathogen	Respondents (n)
No idea	21 (13.7%)
Gram-positive cocci	85 (55.6%)
Gram-negative cocci	14 (9.1%)
Gram-positive bacillus	20 (13.1%)
Gram-negative bacillus	13 (8.5%)
Total	153

Significantly more respondents from the referral eye hospital (92.7%) than respondents from the primary hospital (69.4%) were administered intraocular antibiotics injection for prophylaxis. More intracameral injections (64.7% vs. 35.3%) were used in the referral eye hospital compared to the primary hospital. Moreover, less combined application of antibiotics was used in the referral eye hospital (56.9%) than in the primary hospital (73.5%) (Table 6). However, there were fewer differences from the practice of intraocular antibiotics administration related to the annual volume of primary eye repair surgery (Table 7).

**Table 6:** Practice of intraocular antibiotics in open globe injuries and hospital type.

	Referral eye hospital	Primary hospital	*P*-value
Use of prophylactic intraocular antibiotics			
Routinely use	17 (30.9%)	15 (15.3%)	
Depend on different conditions	34 (61.8%)	53 (54.1%)	
Never use	4 (7.3%)	30 (30.6%)	0.001
Total	55	98	
Method of administration			
Intracameral injection	33 (64.7%)	24 (35.3%)	
Intravitreal injection	15 (29.4%)	35 (51.5%)	
Injection via wound	3 (5.9%)	9 (13.2%)	0.007
Total	51	68	
Choice of antibiotics			
Fluoroquinolones	16 (31.4%)	13 (19.1%)	0.136
Cephalosporin	24 (47.1%)	40 (58.8%)	0.265
Aminoglycoside	9 (17.6%)	7 (10.3%)	0.285
Vancomycin	19 (37.3%)	31 (45.6%)	0.453
Others	0	3 (4.4%)	
Total	51	68	
Combination of antibiotics			
Routinely combine	16 (31.4)	10 (14.7%)	
Depends on different conditions	13 (25.5%)	40 (58.8%)	
Never combine	22 (43.1%)	18 (26.5%)	0.001
Total	51	68	
The most common causative pathogen			
No idea	8(14.5%)	13 (13.3%)	
Gram-positive cocci	28 (50.9%)	57 (58.2%)	
Gram-negative cocci	8 (14.5%)	6 (6.1%)	
Gram-positive bacillus	5 (9.2%)	15 (15.3%)	
Gram-negative bacillus	6 (10.9%)	7 (7.1%)	0.319
Total	55	98	

**Table 7:** Practice of intraocular antibiotics in open globe injuries and the annual eye repair volume.

	0–50	>50	*P*-value
Use of prophylactic intraocular antibiotics			
Routinely use	17 (20.5%)	15 (21.4%)	
Depend on different conditions	50 (60.2%)	37 (52.9%)	
Not use	16 (19.3%)	18 (25.7%)	0.574
Total	83	70	
Method of administration			
Intracameral injection	34 (50.7%)	23(44.2%)	
Intravitreal injection	25 (37.4%)	25 (48.1%)	
Injection via wound	8 (11.9%)	4 (7.7%)	0.456
Total	67	52	
Choice of antibiotics			
Fluoroquinolones	17 (25.4%)	12 (23.1%)	0.832
Cephalosporin	36 (53.7%)	28 (53.8%)	1.0
Aminoglycoside	9 (13.4%)	7 (13.5%)	1.0
Vancomycin	25 (37.3%)	25 (48.1%)	0.265
Others	2 (2.9%)	1 (1.9%)	
Total	67	52	
Combination of antibiotics			
Routinely combine	14 (20.9)	12 (23.1%)	
Depends on different conditions	32 (47.8%)	21 (40.4%)	
Not combine	21 (31.3%)	19 (36.5%)	0.701
Total	67	52	
The most common causative pathogen in post-traumatic endophthalmology			
No idea	14 (16.9%)	7 (10.0%)	
Gram-positive cocci	42 (50.6%)	43 (61.4%)	
Gram-negative cocci	9 (10.8%)	5 (7.1%)	
Gram-positive bacillus	10 (12.0%)	10 (14.3%)	
Gram-negative bacillus	8 (9.6%)	5 (7.1%)	0.558
Total	83	70	

## Discussion

This survey provides the first data on conditions of intraocular antibiotic administration for prophylaxis after open globe injuries in China. In this survey, acquiring data online with smartphones offers convenience over traditional methods, such as telephone interview, postal survey or online survey using a computer. Because the participants have freewill to access this survey, only ophthalmologists interested in this issue would be likely to complete the questionnaire. Thus, we have more confidence to believe the efficiency of this survey in reflecting the actual conditions of intraocular antibiotics for prophylaxis in open globe injuries in China, although the response rate was not very high (30.6%).

Previous studies have reported microorganism cultures from the anterior chamber to be positive in 26%–50.9% of cases of open globe injuries at the conclusion of primary eye repair [23–25], which provides evidence for antibiotic use for endophthalmitis prophylaxis. Intraocular injection can cross the blood-ocular barrier and appears to be the most effective method to achieve a high concentration of intraocular antibiotics. Narang and colleagues have reported that the incidence of endophthalmitis (6.27%) with 1 mg prophylactic intravitreal vancomycin and 2.25 mg ceftazidime at the end of primary eye repair was lower than with only systemic prophylaxis (18.42%). However, it was not statistically significant [17]. Another clinical trial by Sohelian and colleagues also revealed that the incidence of endophthalmitis (0.8%) with prophylactic intracameral or intravitreal 40-μg gentamicin sulfate and 45 μg clindamycin was lower than with injection of a balanced salt solution (2.3%). However, the benefit of intraocular antibiotics use was only observed in eyes with IOFB [15]. Without data from a larger prospective randomized and long follow-up clinical trial, routinely intraocular antibiotics use in open globe injuries has not been listed in the prophylactic guidelines for post-traumatic endophthalmitis. However, the survey demonstrated that a majority of the respondents had administered intraocular antibiotics for prophylaxis at the end of primary eye repair. In these respondents, 20.9% used these antibiotics routinely, and 56.9% of respondents administered only in selected high-risk cases with open globe injuries. This finding indicated that intraocular antibiotics administration in post-traumatic endophthalmitis prophylaxis is popular in China.

With the decision to administer intraocular antibiotics, 47.9% of respondents choose an intracameral injection in this survey; however, 42.0% of respondents preferred intravitreal injection. With the lack of strong evidence from animal and clinical studies, it is difficult to determine whether intracameral or intravitreal injection is more appropriate in post-traumatic endophthalmitis prophylaxis. In the limited two previous clinical studies, Narang and colleagues choose intravitreal injection in all cases[17], while Sohelian and colleagues preferred intracameral injection only in the eyes with anterior lacerations limited to rectus muscle insertions and with an intact lens capsule, and intravitreal injection in eyes with lacerations limited to or extending the posterior to rectus muscle insertions and eyes with a ruptured lens capsule [15]. Theoretically, both intracameral and intravitreal injections have their own limitations. The ruptured eye is commonly associated with suprachoroidal hemorrhage and retinal detachment, which increases the risk for complications with intravitreal injection. However, the dosage of intracameral injection used in the eye with a ruptured lens capsule may cause an inadequate concentration of intraocular antibiotics to achieve an MIC90 for microbes.

As previously reported, the bacterial spectrum from post-traumatic endophthalmitis is relatively different from post-operative endophthalmitis. Coagulase-negative *staphylococci* is more prevalent in post-operative endophthalmitis (45–48%)[8] than in post-traumatic endophthalmitis (21.5%-26%) [26–28]. However, the prevalence of Gram-negative *bacillus* (15.7%-18.5%) is higher in post-traumatic endophthalmitis [26–28], which is commonly associated with poorer visual prognosis. [29] Despite these findings, coagulase-negative *staphylococci* is still the most common pathogen in post-traumatic endophthalmitis. In this survey, only 55.6% of respondents replied Gram-positive *cocci* as the common causative pathogen in post-traumatic endophthalmitis. Moreover, 13.7% of respondents replied having no idea about this question. This finding indicated a poor understanding of the epidemiology of post-traumatic endophthalmitis for most ophthalmologists in China.

According to the bacterium spectrum of post-traumatic endophthalmitis described above, theoretically, the selected antibiotic regimen for prophylaxis or treatment should provide adequate coverage against both gram-positive and gram-negative organisms in open globe injuries. The combination of antibiotic agents is the main method to increase the coverage of the bacterium spectrum. However, in this survey, only 21.9% of respondents replied using two or more antibiotic agents for post-traumatic endophthalmitis prophylaxis routinely. How to choose the appropriate antibiotic agents is not only based on the coverage of bacterium spectrum, but the intraocular safety of antibiotics should also be considered. In post-traumatic endophthalmitis, intravitreal injections of 1.0 mg/0.1 ml vancomycin and 2.25 mg/0.1 ml ceftazidime are current recommendations for empirical therapy as suggested by the Endophthalmitis Vitrectomy Study (EVS) [1]. However, this guideline may not be appropriate for post-traumatic endophthalmitis prophylaxis. Indeed, there is still no consensus among the guidelines for prophylaxis practice. Thus, the choice of antibiotics for prophylaxis after open globe injuries is not based on strong evidences of clinical studies, but on the personal empirics of each eye clinician. Among the listed antibiotic agents in this survey, cephalosporins (53.8%) and vancomycin (42.0%) were more preferential for use, followed by fluoroquinolones (24.3%) and aminoglycosides (13.4%). Because it provides good efficacy against gram-positive organisms, the first and second generations of cephalosporins are commonly suggested choices for prophylaxis after surgery, such as cefazolin and cefuroxime. Both cefazoline and cefuroxime have an excellent safety profile for intraocular administration. However, the increasing resistance to cefazolin limits its application. Cefuroxime is the first choice for endophthalmitis prophylaxis after cataract surgery in Europe, which revealed that 1.0 mg/0.1 ml intracameral cefuroxime reduced the risk of endophthalmitis after cataract surgery in previous studies [30–32]. Vancomycin is susceptible against all Gram-positive organisms, including methicillin-resistant *Staphylococcus aureus* (MRSA). Intravitreal vancomycin is considered to be the first choice for therapy in endophthalmitis. However, the use of intraocular injection of vancomycin for endophthalmitis prophylaxis still remains more controversial. It has been reported that vancomycin-resistant *enterococci* has previously emerged [33, 34]. The wide use of vancomycin for prophylaxis may result in an increasing resistance to vancomycin. However, our survey indicated the high prevalence (42.0%) of using intraocular vancomycin for post-trauma endophthalmitis prophylaxis in China. Regarding the good antimicrobial coverage against gram-negative organisms, aminoglycosides, such as gentamicin, tobramycin or amikacin, have also been used intravitreally for the treatment and prophylaxis of endophthalmitis [1, 15, 18]. However, it has been reported that retinal toxicity with severe vision loss occurred with intravitreal injection of aminoglycosides, particularly with gentamicin [35]. Thus, intravitreal aminoglycosides are not widely suggested for prophylaxis in the eyes without the establishment of endophthalmitis. Due to its good penetration into the vitreous and excellent antimicrobial coverage against *Bacillus*, fluoroquinolones are widely used topically and systemically for the treatment and prophylaxis of endophthalmitis. Although the safety and efficacy of intraocular injection with fluoroquinolones, such as levofloxacin or moxifloxacin, have been evaluated in experimental endophthalmitis, [36–38] the efficacy of intraocular injection with fluoroquinolones has not been well established in human. However, 24.3% of respondents replied using fluoroquinolones as the first choice in this survey.

Usually, a surgeon with a large volume of surgery or working at a high-level hospital may indicate a favorable clinical practice. In this survey, there were no differences from the practice of intraocular antibiotics administration related to the annual volume of primary eye repair surgery; however, there was a higher prevalence of using intraocular antibiotics for post-traumatic endophthalmitis prophylaxis at the referral eye hospital than at the primary hospital. In China, the educational background of ophthalmologists among the different level hospitals is unequal. Moreover, there are ophthalmologists who specialize on eye trauma only at the referral eye hospital, but not at the primary hospital. These factors may result in the difference in the practice of using intraocular antibiotics between the two types of hospitals.

However, there were a few limitations to our study. Our population was conference-based, therefore, the surveyed participants might not be fully representative of the general population of Chinese ophthalmologists, with lacking of socio-demographic information (e.g., age, gender, area of residence, education background, etc.) of the participants. Secondly, although participants enrolled voluntarily ensured the efficiency of this survey, it might have introduced selection bias.

In conclusion, intraocular antibiotics injection for post-traumatic endophthalmitis prophylaxis has been previously and widely used in China, although most injections were performed only in cases with high risk of endophthalmitis. However, the choice of antibiotic agents and the intraocular route of delivery vary, even a bit beyond common practice. Without well-established clinical evidences, intraocular antibiotic administration routinely for prophylaxis at the end of open globe injuries has not been widely accepted by most ophthalmologists globally. To establish a standardized protocol of intraocular antibiotics administration for post-traumatic endophthalmitis prophylaxis, many questions still need to be answered. For example, which antibiotic agent or combination of antibiotic agents is the most effective? What dosage of antibiotic agents is the safest without potential toxicity? Which route of delivery is in the best risk-benefit ratio? Is it complicated by the alteration of protective normal host flora and increase in antimicrobial resistance? Thus, a large prospective randomized and multi-centered clinical trial with long follow-up should be performed immediately.

## Supporting Information

S1 File The questionnaire for this survey. (PDF)

S2 File All original data in this study. (PDF)
