# Supplementary material for: Survey of Intraocular Antibiotics Prophylaxis Practice after Open Globe Injury in China
Source: PLoS One. 2016 Jun 8;11(6):e0156856. doi: 10.1371/journal.pone.0156856 (PMC4898702; doi:10.1371/journal.pone.0156856)
Supplement: S1 File — (PDF) [file pone.0156856.s001.pdf]

Questionnair regarding to intraocular antibiotics use in open globe injuries

1. Which hospital are you from?

A. referral eye hospital B. primary hospital

2. How many primary eye repair surgeries do you perform annually?

A. 0 case B. 1~50 cases C. 50~100 cases D. > 100 cases

3. Do you administer intraocular antibiotics at the end the primary eye repair?

A. routinely use B. do not use

C. depends on different conditions (check all that apply)

a. rupture of lens capsule

b. intraocular foreign body remain

c. delayed primary closure of the wound >24 hours

d. injured in dirty environment

e. serious inflammation reaction

f. huge wound

g. other

4. How do you administrate antibiotic agents into an eye?

A. intracameral injection B. intravitreal injection C. injection via wound

5. What kind of antibiotic agent is your first choice for intraocular administration? (check all that apply)

A. fluoroquinolones B. cephalosporin C. aminoglycosides D. vancomycin E. others

6. Do you prefer combination of two or more than two intraocular antibiotic agents?

A. routinely use B. depends on different conditions C. do not use

7. Why do you prefer combination of intraocular antibiotic agents?

A. reduce antimicrobial resistance B. increase antimicrobial efficiency

C .increase antimicrobial spectrum coverage

8. In your opinion, which is the most common causative pathogen in post-traumatic endophthalmitis?

A. no idea B. Gram-positive cocci C. Gram-negative cocci

D. Gram-positve bacillus E. Gram-negative bacillus
