# Supplementary material for: Survey of Intraocular Antibiotics Prophylaxis Practice after Open Globe Injury in China
Source: PLoS One. 2016 Jun 8;11(6):e0156856. doi: 10.1371/journal.pone.0156856 (PMC4898702; doi:10.1371/journal.pone.0156856)
Supplement: S2 File — (PDF) [file pone.0156856.s002.pdf]

开始时间	结束时间	状态	Q1_您来自什么医院_眼	Q1_您来自什么	Q2_您每年
2014/10/30 14:28	2014/10/30 14:31	正常完成	是	是	50~100
2014/10/30 14:34	2014/10/30 14:37	正常完成	否	是	0~50
2014/10/30 15:42	2014/10/30 15:46	正常完成	否	否	＞100
2014/10/30 15:42	2014/10/30 15:47	正常完成	是	是	0~50
2014/10/30 15:42	2014/10/30 15:47	正常完成	否	是	0~50
2014/10/30 15:42	2014/10/30 15:51	正常完成	是	否	0~50
2014/10/30 16:12	2014/10/30 16:15	正常完成	否	是	＞100
2014/10/30 16:41	2014/10/30 16:48	正常完成	是	是	＞100
2014/10/30 16:44	2014/10/30 16:48	正常完成	是	是	0~50
2014/10/30 16:42	2014/10/30 16:48	正常完成	是	是	＞100
2014/10/30 16:46	2014/10/30 16:50	正常完成	是	是	＞100
2014/10/30 16:51	2014/10/30 16:54	正常完成	是	是	50~100
2014/10/30 16:56	2014/10/30 17:00	正常完成	是	否	0~50
2014/10/30 17:00	2014/10/30 17:03	正常完成	是	否	0~50
2014/10/30 16:58	2014/10/30 17:04	正常完成	是	是	0~50
2014/10/30 17:00	2014/10/30 17:07	正常完成	是	是	0~50
2014/10/30 17:13	2014/10/30 17:16	正常完成	否	否	50~100
2014/10/30 17:20	2014/10/30 17:23	正常完成	否	否	0~50
2014/10/30 17:40	2014/10/30 17:42	正常完成	是	是	＞100
2014/10/30 17:43	2014/10/30 17:46	正常完成	是	是	＞100
2014/10/30 17:45	2014/10/30 17:47	正常完成	是	是	＞100
2014/10/30 17:42	2014/10/30 17:47	正常完成	否	是	50~100
2014/10/30 17:46	2014/10/30 17:47	正常完成	否	是	50~100
2014/10/30 17:46	2014/10/30 17:48	正常完成	是	是	0~50
2014/10/30 17:45	2014/10/30 17:49	正常完成	否	否	＞100
2014/10/30 17:50	2014/10/30 17:54	正常完成	是	是	50~100
2014/10/30 17:53	2014/10/30 17:56	正常完成	否	是	50~100
2014/10/30 17:54	2014/10/30 17:59	正常完成	是	是	＞100
2014/10/30 18:01	2014/10/30 18:03	正常完成	否	是	0~50
2014/10/30 18:21	2014/10/30 18:24	正常完成	否	否	0~50
2014/10/30 19:42	2014/10/30 19:45	正常完成	是	是	＞100
2014/10/30 21:48	2014/10/30 21:50	正常完成	是	是	0~50
2014/10/31 8:46	2014/10/31 8:49	正常完成	是	是	0~50
2014/10/31 8:48	2014/10/31 8:50	正常完成	是	是	50~100
2014/10/31 8:53	2014/10/31 8:56	正常完成	是	否	50~100
2014/10/31 8:59	2014/10/31 9:03	正常完成	否	是	0~50
2014/10/31 9:07	2014/10/31 9:10	正常完成	否	是	＞100
2014/10/31 9:07	2014/10/31 9:11	正常完成	是	否	0~50
2014/10/31 9:07	2014/10/31 9:11	正常完成	是	是	0~50
2014/10/31 9:06	2014/10/31 9:12	正常完成	否	是	50~100
2014/10/31 9:07	2014/10/31 9:12	正常完成	是	否	0~50
2014/10/31 9:13	2014/10/31 9:15	正常完成	否	是	0~50
2014/10/31 9:45	2014/10/31 9:54	正常完成	否	是	50~100
2014/10/31 9:53	2014/10/31 9:58	正常完成	否	否	0~50
2014/10/31 10:02	2014/10/31 10:06	正常完成	否	是	0~50
2014/10/31 10:05	2014/10/31 10:11	正常完成	是	否	0~50
2014/10/31 10:17	2014/10/31 10:19	正常完成	是	是	0~50
2014/10/31 10:22	2014/10/31 10:24	正常完成	是	是	0~50
2014/10/31 10:26	2014/10/31 10:28	正常完成	是	否	＞100
2014/10/31 10:26	2014/10/31 10:30	正常完成	是	是	50~100
2014/10/31 10:34	2014/10/31 10:37	正常完成	否	是	0~50
2014/10/31 10:42	2014/10/31 10:44	正常完成	是	是	0~50
2014/10/31 10:45	2014/10/31 10:47	正常完成	是	否	0~50

2014/10/31 10:44	2014/10/31 10:47	正常完成	是	是	0~50
2014/10/31 10:50	2014/10/31 10:52	正常完成	是	否	0~50
2014/10/31 10:50	2014/10/31 10:52	正常完成	是	否	0~50
2014/10/31 10:52	2014/10/31 10:54	正常完成	否	是	50~100
2014/10/31 10:53	2014/10/31 10:57	正常完成	是	否	0~50
2014/10/31 11:11	2014/10/31 11:14	正常完成	否	是	50~100
2014/10/31 11:14	2014/10/31 11:15	正常完成	是	是	0~50
2014/10/31 11:54	2014/10/31 11:56	正常完成	是	是	50~100
2014/10/31 11:55	2014/10/31 11:58	正常完成	否	是	0~50
2014/10/31 11:55	2014/10/31 11:59	正常完成	是	是	＞100
2014/10/31 11:59	2014/10/31 12:01	正常完成	否	是	0~50
2014/10/31 11:59	2014/10/31 12:01	正常完成	是	是	＞100
2014/10/31 12:05	2014/10/31 12:06	正常完成	否	是	0~50
2014/10/31 12:07	2014/10/31 12:11	正常完成	是	是	0~50
2014/10/31 12:07	2014/10/31 12:12	正常完成	否	是	50~100
2014/10/31 12:22	2014/10/31 12:25	正常完成	是	是	50~100
2014/10/31 12:36	2014/10/31 12:38	正常完成	是	是	50~100
2014/10/31 12:29	2014/10/31 12:46	正常完成	否	是	＞100
2014/10/31 12:47	2014/10/31 12:50	正常完成	是	否	50~100
2014/10/31 12:49	2014/10/31 12:51	正常完成	否	是	0~50
2014/10/31 12:29	2014/10/31 12:53	正常完成	否	是	＞100
2014/10/31 12:53	2014/10/31 12:55	正常完成	是	否	50~100
2014/10/31 12:56	2014/10/31 13:00	正常完成	否	否	0~50
2014/10/31 13:19	2014/10/31 13:23	正常完成	否	是	0~50
2014/10/31 13:19	2014/10/31 13:24	正常完成	否	是	0~50
2014/10/31 13:19	2014/10/31 13:24	正常完成	否	是	0~50
2014/10/31 13:31	2014/10/31 13:36	正常完成	是	否	0~50
2014/10/31 13:41	2014/10/31 13:45	正常完成	是	否	0~50
2014/10/31 13:42	2014/10/31 13:46	正常完成	否	否	50~100
2014/10/31 13:45	2014/10/31 13:47	正常完成	否	是	0~50
2014/10/31 13:46	2014/10/31 13:49	正常完成	否	是	0~50
2014/10/31 13:46	2014/10/31 13:50	正常完成	否	否	50~100
2014/10/31 13:48	2014/10/31 13:51	正常完成	是	否	0~50
2014/10/31 13:50	2014/10/31 13:53	正常完成	是	是	0~50
2014/10/31 13:51	2014/10/31 13:55	正常完成	否	否	0~50
2014/10/31 13:59	2014/10/31 14:04	正常完成	是	是	0~50
2014/10/31 14:03	2014/10/31 14:06	正常完成	是	否	＞100
2014/10/31 14:04	2014/10/31 14:06	正常完成	是	否	0~50
2014/10/31 14:12	2014/10/31 14:13	正常完成	否	是	0~50
2014/10/31 14:24	2014/10/31 14:26	正常完成	否	是	＞100
2014/10/31 14:40	2014/10/31 14:42	正常完成	否	否	50~100
2014/10/31 14:41	2014/10/31 14:42	正常完成	是	是	50~100
2014/10/31 15:13	2014/10/31 15:16	正常完成	是	否	0~50
2014/10/31 15:39	2014/10/31 15:43	正常完成	是	否	＞100
2014/10/31 15:51	2014/10/31 15:54	正常完成	是	是	50~100
2014/10/31 15:51	2014/10/31 15:55	正常完成	是	是	50~100
2014/10/31 15:57	2014/10/31 16:00	正常完成	是	否	50~100
2014/10/31 15:57	2014/10/31 16:00	正常完成	是	否	0~50
2014/10/31 16:00	2014/10/31 16:01	正常完成	是	是	0~50
2014/10/31 16:09	2014/10/31 16:12	正常完成	否	否	0~50
2014/10/31 16:13	2014/10/31 16:16	正常完成	否	是	0~50
2014/10/31 16:15	2014/10/31 16:27	正常完成	否	是	0~50
2014/10/31 16:29	2014/10/31 16:32	正常完成	是	否	0~50
2014/10/31 16:31	2014/10/31 16:34	正常完成	否	是	50~100

2014/10/31 16:32	2014/10/31 16:35	正常完成	是	否	0~50
2014/10/31 16:39	2014/10/31 16:42	正常完成	是	是	50~100
2014/10/31 16:40	2014/10/31 16:44	正常完成	否	是	＞100
2014/10/31 16:42	2014/10/31 16:46	正常完成	否	是	50~100
2014/10/31 16:49	2014/10/31 16:53	正常完成	否	是	＞100
2014/10/31 17:02	2014/10/31 17:05	正常完成	否	是	0~50
2014/10/31 17:28	2014/10/31 17:30	正常完成	是	是	0~50
2014/10/31 17:38	2014/10/31 17:41	正常完成	是	是	＞100
2014/10/31 17:39	2014/10/31 17:44	正常完成	是	否	50~100
2014/10/31 17:42	2014/10/31 17:45	正常完成	是	是	0~50
2014/10/31 17:44	2014/10/31 17:46	正常完成	是	是	0~50
2014/10/31 17:38	2014/10/31 17:47	正常完成	否	是	50~100
2014/10/31 17:45	2014/10/31 17:47	正常完成	是	是	0~50
2014/10/31 17:44	2014/10/31 17:49	正常完成	是	否	0~50
2014/10/31 19:37	2014/10/31 19:38	正常完成	否	是	0~50
2014/10/31 20:43	2014/10/31 20:46	正常完成	否	是	50~100
2014/10/31 22:25	2014/10/31 22:29	正常完成	是	是	50~100
2014/10/31 22:43	2014/10/31 22:45	正常完成	是	否	50~100
2014/10/31 22:45	2014/10/31 22:49	正常完成	是	是	0~50
2014/10/31 23:25	2014/10/31 23:33	正常完成	是	是	50~100
2014/11/1 8:24	2014/11/1 8:26	正常完成	否	否	0~50
2014/11/1 8:50	2014/11/1 8:52	正常完成	否	是	50~100
2014/11/1 9:13	2014/11/1 9:18	正常完成	否	是	0~50
2014/11/1 9:49	2014/11/1 9:51	正常完成	否	否	0~50
2014/11/1 10:05	2014/11/1 10:07	正常完成	是	是	0~50
2014/11/1 10:05	2014/11/1 10:08	正常完成	是	是	0~50
2014/11/1 10:05	2014/11/1 10:08	正常完成	是	否	0~50
2014/11/1 10:06	2014/11/1 10:16	正常完成	否	是	0~50
2014/11/1 11:06	2014/11/1 11:07	正常完成	否	否	0~50
2014/11/1 13:03	2014/11/1 13:09	正常完成	否	是	＞100
2014/11/1 13:29	2014/11/1 13:33	正常完成	否	是	＞100
2014/11/1 13:32	2014/11/1 13:40	正常完成	否	是	50~100
2014/11/1 14:23	2014/11/1 14:25	正常完成	否	是	50~100
2014/11/1 18:55	2014/11/1 19:06	正常完成	否	是	＞100
2014/11/2 18:24	2014/11/2 18:27	正常完成	否	是	0~50
2014/11/2 22:42	2014/11/2 22:45	正常完成	是	是	50~100
2014/11/3 4:16	2014/11/3 4:19	正常完成	是	是	0~50
2014/11/3 13:23	2014/11/3 13:26	正常完成	是	是	50~100
2014/11/3 13:24	2014/11/3 13:26	正常完成	是	是	0~50
2014/11/3 13:32	2014/11/3 13:33	正常完成	否	否	0~50
2014/11/3 13:41	2014/11/3 13:45	正常完成	否	是	50~100
2014/11/3 14:01	2014/11/3 14:05	正常完成	是	否	50~100
2014/11/3 14:12	2014/11/3 14:15	正常完成	是	是	0~50
2014/11/3 15:14	2014/11/3 15:22	正常完成	否	否	0~50
2014/11/3 21:22	2014/11/3 21:30	正常完成	否	否	0~50
2014/11/3 21:56	2014/11/3 22:01	正常完成	否	是	50~100

Q3_您是否给开放性眼外伤	Q4_您在何种情况下会选择	Q4_您在何	Q4_您在何	Q4_您在何	Q4_您在何
视情况不同，有时候用			伤口延迟修补＞24小时		
视情况不同，有时候用	晶状体囊膜破裂				炎症反应
基本不做抗生素眼内注药					
视情况不同，有时候用		眼内异物	伤口延迟	在污染环	炎症反应
视情况不同，有时候用		眼内异物		在污染环境受伤	
视情况不同，有时候用				在污染环	炎症反应
视情况不同，有时候用		眼内异物			
视情况不同，有时候用	晶状体囊膜破裂	眼内异物	伤口延迟	在污染环境受伤	
视情况不同，有时候用			伤口延迟	在污染环境受伤	
视情况不同，有时候用	晶状体囊膜破裂	眼内异物	伤口延迟	在污染环	炎症反应
视情况不同，有时候用		眼内异物	伤口延迟	在污染环	炎症反应
常规行眼内抗生素注药					
视情况不同，有时候用				在污染环	炎症反应
基本不做抗生素眼内注药					
视情况不同，有时候用	晶状体囊膜破裂	眼内异物			
常规行眼内抗生素注药					
基本不做抗生素眼内注药					
视情况不同，有时候用		眼内异物			炎症反应
视情况不同，有时候用				在污染环	炎症反应
视情况不同，有时候用		眼内异物	伤口延迟	在污染环境受伤	
视情况不同，有时候用	晶状体囊膜破裂	眼内异物	伤口延迟	在污染环	炎症反应
基本不做抗生素眼内注药					
基本不做抗生素眼内注药					
视情况不同，有时候用		眼内异物			
视情况不同，有时候用	晶状体囊膜破裂			在污染环	炎症反应
常规行眼内抗生素注药					
常规行眼内抗生素注药					
视情况不同，有时候用					炎症反应
视情况不同，有时候用		眼内异物			
基本不做抗生素眼内注药					
视情况不同，有时候用		眼内异物	伤口延迟	在污染环	炎症反应
视情况不同，有时候用		眼内异物		在污染环	炎症反应
常规行眼内抗生素注药					
视情况不同，有时候用	晶状体囊膜破裂				
视情况不同，有时候用		眼内异物			
视情况不同，有时候用		眼内异物	伤口延迟	在污染环境受伤	
视情况不同，有时候用				在污染环	炎症反应
视情况不同，有时候用				在污染环境受伤	
视情况不同，有时候用				在污染环境受伤	
视情况不同，有时候用	晶状体囊膜破裂	眼内异物	伤口延迟	在污染环	炎症反应
视情况不同，有时候用		眼内异物		在污染环境受伤	
视情况不同，有时候用	晶状体囊膜破裂	眼内异物	伤口延迟修补＞24小时		
基本不做抗生素眼内注药					
视情况不同，有时候用			伤口延迟	在污染环	炎症反应
视情况不同，有时候用		眼内异物	伤口延迟修补＞24小		炎症反应
常规行眼内抗生素注药					
基本不做抗生素眼内注药					
常规行眼内抗生素注药					
常规行眼内抗生素注药					
常规行眼内抗生素注药					
视情况不同，有时候用	晶状体囊膜破裂		伤口延迟	在污染环	炎症反应
常规行眼内抗生素注药					
常规行眼内抗生素注药					

视情况不同，有时候用			伤口延迟修补＞24小时		
基本不做抗生素眼内注药					
视情况不同，有时候用	晶状体囊膜破裂	眼内异物			炎症反应重
视情况不同，有时候用			伤口延迟修补＞24小时		
视情况不同，有时候用					炎症反应重
基本不做抗生素眼内注药					
常规行眼内抗生素注药					
常规行眼内抗生素注药					
视情况不同，有时候用					炎症反应重
视情况不同，有时候用					炎症反应重
常规行眼内抗生素注药					
常规行眼内抗生素注药					
常规行眼内抗生素注药					
视情况不同，有时候用					炎症反应重
视情况不同，有时候用			伤口延迟修	在污染环境受伤	
基本不做抗生素眼内注药					
视情况不同，有时候用		眼内异物			
视情况不同，有时候用	晶状体囊膜破裂	眼内异物	伤口延迟修	在污染环境	炎症反应重
视情况不同，有时候用		眼内异物	伤口延迟修	在污染环境受伤	
视情况不同，有时候用					炎症反应重
基本不做抗生素眼内注药					
常规行眼内抗生素注药					
视情况不同，有时候用		眼内异物	伤口延迟修	在污染环境	炎症反应重
视情况不同，有时候用	晶状体囊膜破裂	眼内异物	伤口延迟修补＞24小		炎症反应重
视情况不同，有时候用		眼内异物		在污染环境	炎症反应重
视情况不同，有时候用		眼内异物	伤口延迟修补＞24小时		
视情况不同，有时候用		眼内异物	伤口延迟修	在污染环境	炎症反应重
基本不做抗生素眼内注药					
视情况不同，有时候用			伤口延迟修	在污染环境	炎症反应重
常规行眼内抗生素注药					
视情况不同，有时候用			伤口延迟修	在污染环境	炎症反应重
常规行眼内抗生素注药					
基本不做抗生素眼内注药					
视情况不同，有时候用			伤口延迟修	在污染环境受伤	
视情况不同，有时候用	晶状体囊膜破裂	眼内异物		在污染环境受伤	
常规行眼内抗生素注药					
常规行眼内抗生素注药					
基本不做抗生素眼内注药					
视情况不同，有时候用			伤口延迟修补＞24小时		
常规行眼内抗生素注药					
常规行眼内抗生素注药					
常规行眼内抗生素注药					
基本不做抗生素眼内注药					
基本不做抗生素眼内注药					
常规行眼内抗生素注药					
视情况不同，有时候用			伤口延迟修补＞24小时		
基本不做抗生素眼内注药					
常规行眼内抗生素注药					
常规行眼内抗生素注药					
基本不做抗生素眼内注药					
基本不做抗生素眼内注药					
视情况不同，有时候用		眼内异物	伤口延迟修	在污染环境	炎症反应重
常规行眼内抗生素注药					
基本不做抗生素眼内注药					

视情况不同，有时候用				在污染环境受伤	
视情况不同，有时候用		眼内异物			
基本不做抗生素眼内注药					
视情况不同，有时候用		眼内异物	伤口延迟	在污染环	炎症反应
常规行眼内抗生素注药					
视情况不同，有时候用		眼内异物			炎症反应
常规行眼内抗生素注药					
视情况不同，有时候用		眼内异物		在污染环	炎症反应
视情况不同，有时候用				在污染环境受伤	
视情况不同，有时候用	晶状体囊膜破裂				
视情况不同，有时候用		眼内异物	伤口延迟	在污染环	炎症反应
视情况不同，有时候用		眼内异物			
视情况不同，有时候用					炎症反应
视情况不同，有时候用		眼内异物		在污染环	炎症反应
视情况不同，有时候用	晶状体囊膜破裂				
基本不做抗生素眼内注药					
视情况不同，有时候用		眼内异物	伤口延迟	在污染环	炎症反应
基本不做抗生素眼内注药					
视情况不同，有时候用		眼内异物	伤口延迟	在污染环	炎症反应
视情况不同，有时候用		眼内异物		在污染环	炎症反应
基本不做抗生素眼内注药					
视情况不同，有时候用		眼内异物	伤口延迟	在污染环境受伤	
基本不做抗生素眼内注药					
常规行眼内抗生素注药					
视情况不同，有时候用		眼内异物	伤口延迟	在污染环	炎症反应
常规行眼内抗生素注药					
视情况不同，有时候用		眼内异物	伤口延迟	在污染环	炎症反应
基本不做抗生素眼内注药					
视情况不同，有时候用	晶状体囊膜破裂				
视情况不同，有时候用		眼内异物			炎症反应
视情况不同，有时候用		眼内异物		在污染环境受伤	
视情况不同，有时候用	晶状体囊膜破裂	眼内异物	伤口延迟	在污染环	炎症反应
基本不做抗生素眼内注药					
视情况不同，有时候用		眼内异物	伤口延迟	在污染环	炎症反应
基本不做抗生素眼内注药					
视情况不同，有时候用		眼内异物		在污染环	炎症反应
视情况不同，有时候用	晶状体囊膜破裂	眼内异物	伤口延迟	在污染环	炎症反应
常规行眼内抗生素注药					
视情况不同，有时候用		眼内异物		在污染环	炎症反应
基本不做抗生素眼内注药					
基本不做抗生素眼内注药					
基本不做抗生素眼内注药					
基本不做抗生素眼内注药					
视情况不同，有时候用		眼内异物	伤口延迟	在污染环	炎症反应
视情况不同，有时候用		眼内异物		在污染环	炎症反应
视情况不同，有时候用		眼内异物	伤口延迟	在污染环	炎症反应

Q4_您在何	Q4_您在何	Q4_您在何	Q5_您做一	Q6_您常用	Q6_您常用	Q6_您常用	Q6_您常用	Q6_您常用
			经玻璃体	喹诺酮				
重			经伤口注药		氨基糖甙	头孢类		
大伤口			经伤口注药			头孢类	万古霉素	
大伤口			经前房注药			头孢类		
重			经玻璃体腔注药				万古霉素	
			经玻璃体腔注药				万古霉素	
大伤口			经前房注药			头孢类		
大伤口			经前房注药			头孢类		
大伤口			经前房注药			头孢类		
重			经前房注药			头孢类	万古霉素	
			经伤口注	喹诺酮				
重			经伤口注药			头孢类		
			经玻璃体腔注药		氨基糖甙			
			经前房注	喹诺酮				
重			经伤口注药					其他，可
大伤口			经前房注	喹诺酮		头孢类		
			经玻璃体腔注药			头孢类	万古霉素	
大伤口			经前房注药		氨基糖甙	头孢类	万古霉素	
			经前房注药				万古霉素	
大伤口			经伤口注药			头孢类	万古霉素	
			经前房注药			头孢类		
			经前房注	喹诺酮	氨基糖甙	头孢类		
重			经前房注药			头孢类		
			经玻璃体腔注药			头孢类		
重			经玻璃体腔注药				万古霉素	
重			经玻璃体腔注药				万古霉素	
			经前房注药			头孢类		
			经玻璃体腔注药		氨基糖甙			
			经玻璃体腔注药					其他，可
			经前房注药			头孢类	万古霉素	
重			经玻璃体腔注药			头孢类	万古霉素	
			经伤口注药			头孢类		
			经前房注	喹诺酮				
大伤口			经玻璃体腔注药		氨基糖甙		万古霉素	
大伤口			经玻璃体腔注药			头孢类		
			经玻璃体腔注药			头孢类		
重			经玻璃体腔注药			头孢类		
重			经前房注药			头孢类		
			经前房注药			头孢类		
			经前房注	喹诺酮				
			经前房注药			头孢类		
			经前房注	喹诺酮				
大伤口			经玻璃体	喹诺酮	氨基糖甙	头孢类		
			经前房注药			头孢类		
			经前房注	喹诺酮		头孢类		

	经前房注药				万古霉素
大伤口	经玻璃体腔注药			头孢类	万古霉素
	经玻璃体腔注药				万古霉素
重	经前房注药			头孢类	
	经前房注	喹诺酮			
	经前房注	喹诺酮			
重	经玻璃体腔注药				万古霉素
大伤口	经前房注药		氨基糖甙		
	经玻璃体腔注药			头孢类	
	经玻璃体腔注药			头孢类	
	经玻璃体腔注药				万古霉素
重	经前房注药				万古霉素
	经玻璃体腔注药			头孢类	万古霉素
	经玻璃体	喹诺酮			
大伤口	经玻璃体腔注药				万古霉素
	经玻璃体腔注药				万古霉素
重	经玻璃体腔注药				万古霉素
大伤口	经前房注	喹诺酮		头孢类	
大伤口	经玻璃体腔注药				万古霉素
重	经前房注药				万古霉素
	经前房注药			头孢类	
大伤口	经伤口注药				万古霉素
重	经玻璃体	喹诺酮		头孢类	万古霉素
	经玻璃体	喹诺酮		头孢类	
重	经前房注	喹诺酮			
	经前房注药			头孢类	
大伤口	经伤口注	喹诺酮			
	经前房注	喹诺酮	氨基糖甙	头孢类	
	经前房注药			头孢类	万古霉素
	经前房注药				万古霉素
	经玻璃体腔注药		氨基糖甙		
	经前房注	喹诺酮			
	经玻璃体	喹诺酮	氨基糖甙		
	经前房注药			头孢类	
	经前房注	喹诺酮			
	经前房注药		氨基糖甙		
	经伤口注	喹诺酮			
	经前房注药		氨基糖甙		
大伤口	经玻璃体腔注药			头孢类	万古霉素
	经前房注药			头孢类	万古霉素

	经前房注药			头孢类		
	经玻璃体腔注药			头孢类		
重	经玻璃体腔注药			头孢类	万古霉素	
	经前房注纟	喹诺酮				
重	经玻璃体腔注药			头孢类	万古霉素	
	经前房注纟	喹诺酮				
大伤口	经玻璃体腔注药			头孢类	万古霉素	
	经玻璃体腔注药			头孢类		
	经玻璃体腔注药				万古霉素	
大伤口	经玻璃体月	喹诺酮	氨基糖甙	头孢类	万古霉素	
	经玻璃体腔注药				万古霉素	
重	经玻璃体腔注药			头孢类		
重	经玻璃体腔注药				万古霉素	
	经前房注药			头孢类		
重	经前房注药				万古霉素	
大伤口	经前房注药			头孢类	万古霉素	
大伤口	经玻璃体腔注药			头孢类	万古霉素	
	经玻璃体腔注药			头孢类	万古霉素	
	经玻璃体腔注药					其他，可歹
大伤口	经玻璃体月	喹诺酮				
	经前房注纟	喹诺酮	氨基糖甙			
大伤口	经前房注药			头孢类		
	经前房注纟	喹诺酮				
重	经玻璃体腔注药			头孢类	万古霉素	
	经玻璃体腔注药			头孢类	万古霉素	
大伤口	经前房注药			头孢类		
大伤口	经伤口注药				万古霉素	
重	经前房注药				万古霉素	
大伤口	经前房注药			头孢类	万古霉素	
	经前房注药			头孢类		
重	经前房注药		氨基糖甙		万古霉素	
大伤口	经前房注药				万古霉素	
重	经玻璃体腔注药			头孢类		
重	经伤口注药			头孢类	万古霉素	

Q6_您常用	Q7_您是否	Q8_您联用	Q8_您联用	Q9_您知道外伤后眼内炎最常见的致病菌是什么吗？
	不会	增加抗菌谱		革兰氏阳性球菌
	视情况不	不经常联用多种抗生		革兰氏阴性球菌
				革兰氏阳性杆菌
	视情况不	增加抗菌效力		革兰氏阳性球菌
	不会	不经常联用多种抗生		格兰仕阴性杆菌
	视情况不	增加抗菌谱		革兰氏阳性杆菌
	会	增加抗菌谱		革兰氏阳性球菌
	不会	增加抗菌谱		革兰氏阳性球菌
	视情况不	增加抗菌效力		革兰氏阳性球菌
	不会	增加抗菌谱		革兰氏阳性球菌
	不会	增加抗菌谱		革兰氏阳性球菌
	视情况不	不经常联用多种抗生		革兰氏阳性球菌
	视情况不	增加抗菌效力		格兰仕阴性杆菌
				革兰氏阳性球菌
	视情况不	增加抗菌效力		革兰氏阴性球菌
	会	不经常联用多种抗生		革兰氏阳性杆菌
				革兰氏阳性球菌
刂举	视情况不	减少耐药		不知道
	视情况不	增加抗菌效力		革兰氏阳性球菌
	视情况不	增加抗菌谱		革兰氏阳性球菌
	不会	增加抗菌谱		革兰氏阳性球菌
				革兰氏阳性球菌
				革兰氏阳性球菌
	不会	不经常联用多种抗生		革兰氏阳性球菌
	视情况不	增加抗菌效力		格兰仕阴性杆菌
	视情况不	增加抗菌效力		革兰氏阴性球菌
	视情况不	不经常联用多种抗生		革兰氏阳性球菌
	视情况不	增加抗菌谱		不知道
	视情况不	不经常联用多种抗生		不知道
				不知道
	视情况不	增加抗菌效力		革兰氏阴性球菌
	视情况不	增加抗菌谱		革兰氏阳性球菌
	会	不经常联用多种抗生		不知道
	视情况不	其他，请说明		革兰氏阳性球菌
刂举	不会	增加抗菌效力		革兰氏阳性杆菌
	不会	增加抗菌谱		革兰氏阳性球菌
	视情况不	增加抗菌谱		革兰氏阳性球菌
	不会	不经常联用多种抗生		格兰仕阴性杆菌
	视情况不	不经常联用多种抗生		格兰仕阴性杆菌
	会	增加抗菌效力		革兰氏阳性球菌
	视情况不	不经常联用多种抗生		革兰氏阴性球菌
	不会	增加抗菌效力		不知道
				革兰氏阴性球菌
	视情况不	减少耐药		革兰氏阳性球菌
	视情况不	减少耐药		革兰氏阳性球菌
	不会	增加抗菌效力		格兰仕阴性杆菌
				不知道
	会	不经常联用多种抗生		格兰仕阴性杆菌
	会	不经常联用多种抗生		革兰氏阳性球菌
	不会	不经常联用多种抗生		革兰氏阳性球菌
	视情况不	增加抗菌谱		革兰氏阳性球菌
	不会	增加抗菌谱		革兰氏阳性杆菌
	会	不经常联用多种抗生		革兰氏阳性球菌

视情况不	增加抗菌效力	革兰氏阴性球菌
		不知道
视情况不	不经常联用多种抗生	革兰氏阳性球菌
视情况不	增加抗菌效力	革兰氏阳性杆菌
视情况不	增加抗菌效力	革兰氏阳性球菌
		格兰仕阴性杆菌
不会	不经常联用多种抗生	革兰氏阳性球菌
不会	不经常联用多种抗生	革兰氏阳性杆菌
不会	增加抗菌效力	革兰氏阳性球菌
会	不经常联用多种抗生	格兰仕阴性杆菌
视情况不	增加抗菌效力	革兰氏阳性球菌
会	增加抗菌谱	革兰氏阳性球菌
不会	增加抗菌效力	革兰氏阳性球菌
视情况不	增加抗菌效力	革兰氏阳性杆菌
视情况不	不经常联用多种抗生	革兰氏阳性球菌
		革兰氏阴性球菌
视情况不	不经常联用多种抗生	革兰氏阳性球菌
不会	增加抗菌谱	革兰氏阳性杆菌
视情况不	增加抗菌效力	革兰氏阳性球菌
不会	增加抗菌效力	革兰氏阳性球菌
		革兰氏阳性球菌
		革兰氏阳性球菌
视情况不	增加抗菌谱	不知道
不会	不经常联用多种抗生	革兰氏阳性球菌
不会	不经常联用多种抗生	革兰氏阳性球菌
视情况不	增加抗菌谱	革兰氏阳性球菌
视情况不	增加抗菌谱	革兰氏阳性球菌
		革兰氏阳性球菌
视情况不	不经常联用多种抗生	革兰氏阳性球菌
会	不经常联用多种抗生	革兰氏阳性杆菌
视情况不	增加抗菌谱	革兰氏阳性杆菌
不会	增加抗菌效力	革兰氏阳性球菌
		革兰氏阳性球菌
视情况不	不经常联用多种抗生	革兰氏阳性球菌
会	减少耐药	革兰氏阳性球菌
视情况不	增加抗菌效力	革兰氏阳性球菌
不会	增加抗菌谱	革兰氏阳性球菌
		革兰氏阳性杆菌
不会	减少耐药	革兰氏阳性球菌
视情况不	不经常联用多种抗生	革兰氏阳性球菌
不会	不经常联用多种抗生	不知道
会	减少耐药	不知道
		不知道
		革兰氏阳性球菌
视情况不	不经常联用多种抗生	不知道
视情况不	增加抗菌效力	革兰氏阳性球菌
		革兰氏阳性球菌
视情况不	不经常联用多种抗生	不知道
会	不经常联用多种抗生	不知道
		革兰氏阳性杆菌
		革兰氏阳性球菌
视情况不	增加抗菌效力	革兰氏阳性球菌
不会	不经常联用多种抗生	不知道
		革兰氏阳性杆菌

	不会	不经常联用多种抗生	革兰氏阴性球菌
	会	增加抗菌谱	不知道
			革兰氏阳性球菌
	视情况不	增加抗菌谱	革兰氏阳性球菌
	会	减少耐药	革兰氏阳性球菌
	会	增加抗菌谱	革兰氏阳性球菌
	会	不经常联用多种抗生	革兰氏阴性球菌
	会	增加抗菌谱	革兰氏阳性杆菌
	视情况不	增加抗菌效力	革兰氏阳性杆菌
	视情况不	不经常联用多种抗生	革兰氏阳性球菌
	会	增加抗菌谱	革兰氏阳性球菌
	不会	增加抗菌效力	革兰氏阳性杆菌
	会	不经常联用多种抗生	革兰氏阴性球菌
	不会	增加抗菌谱	革兰氏阳性球菌
	视情况不	减少耐药	革兰氏阳性杆菌
			革兰氏阳性球菌
	视情况不	增加抗菌谱	格兰仕阴性杆菌
			革兰氏阳性球菌
	不会	不经常联用多种抗生	格兰仕阴性杆菌
	不会	增加抗菌效力	革兰氏阳性球菌
			革兰氏阳性球菌
	会	增加抗菌效力	革兰氏阴性球菌
			革兰氏阳性球菌
刂举	会	增加抗菌效力	不知道
	视情况不	减少耐药	革兰氏阳性球菌
	会	减少耐药	革兰氏阳性球菌
	不会	增加抗菌谱	不知道
			革兰氏阳性球菌
	会	不经常联用多种抗生	革兰氏阴性球菌
	不会	增加抗菌效力	革兰氏阳性球菌
	会	增加抗菌谱	革兰氏阳性球菌
	不会	不经常联用多种抗生	不知道
			革兰氏阳性球菌
	不会	不经常联用多种抗生	不知道
			革兰氏阳性杆菌
	不会	增加抗菌谱	革兰氏阳性球菌
	不会	不经常联用多种抗生	革兰氏阳性球菌
	不会	增加抗菌谱	革兰氏阳性球菌
	不会	增加抗菌谱	格兰仕阴性杆菌
			革兰氏阳性球菌
			革兰氏阳性杆菌
			革兰氏阳性球菌
			革兰氏阴性球菌
	视情况不	增加抗菌效力	革兰氏阳性球菌
	视情况不	增加抗菌效力	革兰氏阳性球菌
	会	增加抗菌谱	格兰仕阴性杆菌
